# Long-Term Impact of Gender Differences After Transcatheter Aortic Valve Implantation

**DOI:** 10.1016/j.cjco.2024.08.012

**Published:** 2024-08-30

**Authors:** Juri Iwata, Kentaro Hayashida, Ryo Arita, Tomonari Moriizumi, Akiyoshi Kajino, Shingo Sakata, Toshinobu Ryuzaki, Keitaro Shinada, Hikaru Tsuruta, Jungo Kato, Tatsuo Takahashi, Masataka Yamazaki, Hideyuki Shimizu, Masaki Ieda

**Affiliations:** a Department of Cardiology, Keio University School of Medicine, Tokyo, Japan; b Department of Anesthesiology, Keio University School of Medicine, Tokyo, Japan; c Department of Cardiovascular Surgery, Keio University School of Medicine, Tokyo, Japan

## Abstract

**Background:**

The short-term and midterm impact of gender differences on transcatheter aortic valve implantation (TAVI) has been studied. However, the impact on long-term clinical outcomes remains unclear. The objective of the study was to investigate the impact of gender differences after TAVI on long-term clinical outcomes and structural valve deterioration (SVD).

**Methods:**

Of the 672 consecutive patients who underwent TAVI, with balloon- or self-expandable valves, between 2013 and 2018, a total of 511 who underwent multidetector computed tomography analysis within 30 days after TAVI were included. Echocardiographic data were analyzed annually.

**Results:**

The number of women was 343 (67.2%), and 90.7% of them had a small annulus (< 430 mm^2^). The effective orifice area was significantly smaller in women compared with that in men, whereas no difference occurred in the incidence of prosthesis–patient mismatch. The incidence of leaflet thrombosis detected by multidetector computed tomography was similar for women vs men (15.2% vs 13.1%, respectively; *P* = 0.53). During the median follow-up of 1844 days (interquartile range: 1190-2311 days), women showed a significantly decreased incidence of all-cause mortality (hazard ratio, 0.69; 95% confidence interval, 0.54-0.90; *P* = 0.005). The development of SVD was comparable (hazard ratio, 0.99; 95% confidence interval, 0.78-1.25, *P* = 0.90). Severe frailty and the balloon-expandable valves were the independent risk factors for all-cause mortality and SVD in women, respectively.

**Conclusions:**

Women had superior long-term clinical outcomes, compared with those of men, despite their having a small annulus. During long-term follow-up, the incidence of SVD in women was similar in the entire cohort, compared to that in men; however, balloon-expandable valves were possible risk factors for SVD in women.

## Graphical abstract

**Figure:**
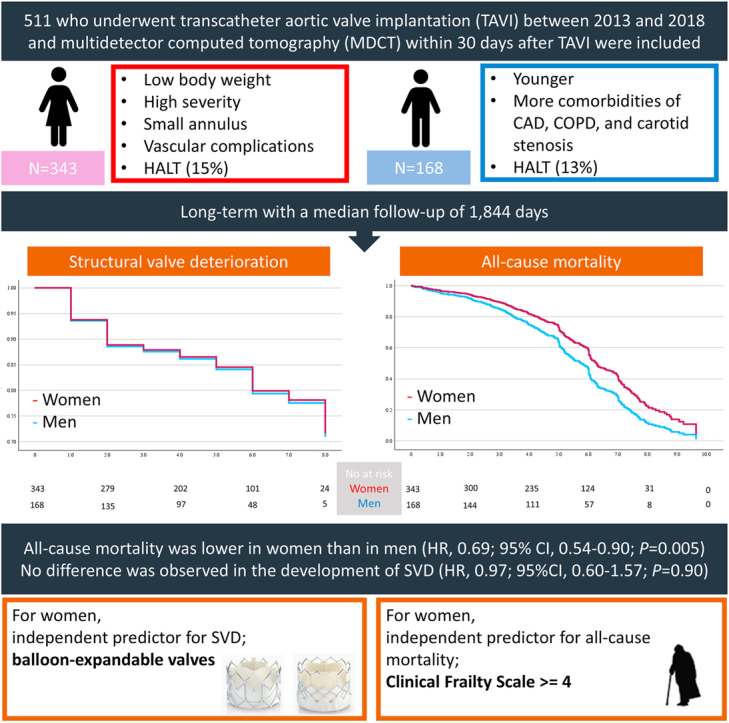

## Résumé

**Contexte:**

Les effets à court et à moyen terme de l'implantation valvulaire aortique par cathéter (IVAC) chez les hommes et chez les femmes ont été étudiés. Cependant, les résultats cliniques à long terme en fonction du sexe demeurent nébuleux. L’étude avait pour objectif d’évaluer dans quelle mesure le sexe a une influence sur les résultats cliniques à long terme et sur la détérioration structurelle de la valve (DSV) après une IVAC.

**Méthodologie:**

Parmi les 672 patients consécutifs qui ont subi une IVAC, par une prothèse valvulaire sur ballonnet ou auto-extensible entre 2013 et 2018, un total de 511 patients qui avaient passé une analyse par tomodensitométrie multidétecteurs au cours des 30 jours suivant l'IVAC ont été retenus. Les données d’échocardiographie ont été analysées sur une base annuelle.

**Résultats:**

Il y avait 343 (67,2 %) femmes; chez 90,7 % d’entre elles, l’anneau était petit (< 430 mm^2^). La surface valvulaire effective était considérablement plus petite chez les femmes que chez les hommes, même si aucune différence n’a été observée quant à la fréquence des incompatibilités prothèse-patient. La fréquence de thrombose valvulaire détectée par tomodensitométrie multidétecteurs était similaire chez les femmes et chez les hommes (respectivement 15,2 % vs 13,1 %; *p* = 0,53). Durant le suivi médian de 1 844 jours (écart interquartile : 1 190-2 311 jours), le taux de mortalité toutes causes confondues a considérablement diminué chez les femmes (rapport de risques instantanés [RRI], 0,69; intervalle de confiance [IC] à 95 % : 0,54-0,90; *p* = 0,005). L’apparition d’une DSV était comparable (RRI, 0,99; IC à 95 % : 0,78-1,25, *p* = 0,90). Une fragilité sévère et des valves sur ballonnet constituaient les facteurs de risques indépendants de mortalité toutes causes confondues et de DSV chez les femmes, respectivement.

**Conclusions:**

Même avec un anneau plus petit, les résultats à long terme étaient supérieurs chez les femmes comparativement aux hommes. Pendant le suivi à long terme, la fréquence de la DSV observée chez les femmes était similaire pour l’ensemble de la cohorte, comparativement à celle observée chez les hommes; toutefois, les valves sur ballonnet représentaient un facteur de risque possible de DSV chez les femmes.

The indications for transcatheter aortic valve implantation (TAVI) have been extended to younger, lower-risk patients.^1^, ^2^, ^3^, ^4^, ^5^ Generally, women have a better prognosis after TAVI if the outcomes are limited to those in midterm data.^6^, ^7^, ^8^, ^9^ However, the long-term prognostic differences based on gender remain unclear. Furthermore, women have a smaller aortic anatomy than men do, owing to their smaller body size; however, the effect of their smaller annulus on valve durability has not been clarified. Therefore, in the era of expanding indications for TAVI, elucidation of gender differences in clinical outcomes and valve durability during long-term follow-up is imperative. Thus, in this study, we aimed to investigate the impact of gender differences on prognosis and structural valve deterioration (SVD) after TAVI, based on long-term follow-up data.

## Methods

### Study cohort and data collection

This study was conducted at the Keio University Hospital. The authors confirm that patient consent forms have been obtained for this article. The Keio University School of Medicine Ethics Committee approved the study protocol (institutional review board information reference numbers: 20130270, UMIN000020423), and this study complied with the principles of the Declaration of Helsinki.

This study included consecutive patients who underwent transfemoral or transapical TAVI using balloon-expandable valves (SAPIEN XT or SAPIEN 3; Edwards Lifesciences, Irvine, CA) or self-expandable valves (CoreValve, Evolut R, or Evolut PRO, Medtronic, Minneapolis, MN) between October 2013 and December 2018 at our centre. We excluded patients who did not undergo multidetector computed tomography (MDCT) within 30 days after TAVI, due to their having either severe chronic kidney disease, allergy to contrast, or asthma, and those who participated in other clinical trials. An institutional Heart Team determined the device selection.

The baseline clinical, procedural, and follow-up data were prospectively captured and recorded in a dedicated database. All procedural complications were defined following the Valve Academic Research Consortium (VARC)-3 criteria and were collected systematically.^10^ Regular follow-ups were scheduled to occur at 1 month, and annually after that. Clinical follow-up data were collected during outpatient visits to our centre, and via documentation from referring physicians, and telephone interviews.

### MDCT analysis

MDCT was performed preoperatively and within 30 days of TAVI. Based on the updated VARC-3 criteria, hypoattenuated leaflet thickening (HALT) was defined as the hypoattenuating thickening of one or more leaflets identified visually using MDCT. The extent of HALT was measured per leaflet, using a 4-tier grading scale for leaflet involvement along a curvilinear contour, assuming maximum involvement from the base of the leaflet.^10^ The presence of HALT was evaluated if at least one grade-1 or greater leaflet was present. Two experienced cardiologists (J.I. and K.H.) evaluated all transcatheter heart valves twice, using contrast-enhanced electrocardiogram-gated MDCT data. All MDCT analyses were performed using 3mensio Structural Heart 10.2 (Pie Medical Imaging, Maastricht, Netherlands).

### Echocardiography and laboratory tests

Transthoracic echocardiography and laboratory tests were performed before the procedure, at discharge, and during the annual follow-up after TAVI. Prosthesis–patient mismatch (PPM) was categorized—based on prosthesis effective orifice area (EOA) indexed to body surface area—as severe (≤ 0.65 cm^2^/m^2^) or moderate (> 0.65-0.85 cm^2^/m^2^) in the nonobese population, and as severe (≤ 0.55 cm^2^/m^2^) or moderate (> 0.55-0.70 cm^2^/m^2^) in the obese population (body mass index ≥ 30 kg/m^2^).^10^ SVD was defined according to the VARC-3 criteria and was categorized as severe (category 1: increase in mean pressure gradient (MPG) ≥ 20 mm Hg, resulting in an MPG ≥ 30 mm Hg, with a concomitant decrease in EOA ≥ 0.6 cm^2^ or ≥ 50%, and/or a decrease in Doppler velocity index of ≥ 0.2 or ≥ 40%; category 2: new occurrence of or increase of ≥ 2 grades of valvular regurgitation, resulting in severe aortic regurgitation) or moderate (category 1: increase in MPG ≥ 10 mm Hg, resulting in an MPG ≥ 20 mm Hg, with a concomitant decrease in EOA ≥ 0.3 cm^2^ or ≥ 25%, and a decrease in the Doppler velocity index of ≥ 0.1 or ≥ 20%; category 2: a new occurrence of or increase of ≥ 1 grade of valvular regurgitation, resulting in increased moderate aortic regurgitation).^10^ In this study, patients with SVD who met the VARC-3 definition of moderate or greater severity were defined as having SVD. The mode of failure was either stenosis or regurgitation. Experienced echocardiographers analyzed the results.

Chronic kidney disease was defined as the presence of an estimated glomerular filtration rate of < 60 mL/min per 1.73 m^2^.

### Endpoint definitions

The main endpoint of this study was all-cause mortality during long-term follow-up after TAVI. Other clinical endpoints, including cardiovascular death, stroke, rehospitalization for heart failure, and SVD development, also were evaluated.

### Statistical analysis

Categorical variables were reported as frequencies and percentages, and differences were evaluated using the χ^2^ test or a 2-tailed Fisher exact test. Continuous variables are presented as mean ± standard deviation, or as median with interquartile range, comparisons between groups were made using the 2-sample *t* test or the Mann–Whitney *U* test, as appropriate. Time-to-event curves were generated using the Kaplan–Meier method (censored at death or last valid contact, awaiting the subsequent follow-up, or consent withdrawal). The Cox proportional hazards model was used to calculate hazard ratios (HRs) and 95% confidence intervals (CIs) of the clinical endpoints. A multivariate logistic regression model was constructed based on the 8 baseline variables that may have affected the study outcomes. The variables included clinical age, HALT levels, body mass index, valve type (balloon-expandable or self-expandable), left ventricular ejection fraction, paravalvular leakage, PPM, clinical frailty scale score, chronic kidney disease, and chronic obstructive pulmonary disease.

Statistical significance was set at a 2-sided *P*-value of < 0.05. All the data analyses were performed using SPSS statistical software, version 29.0 (IBM, Armonk, NY).

## Results

### Study population and baseline characteristics

**Figure 1:**
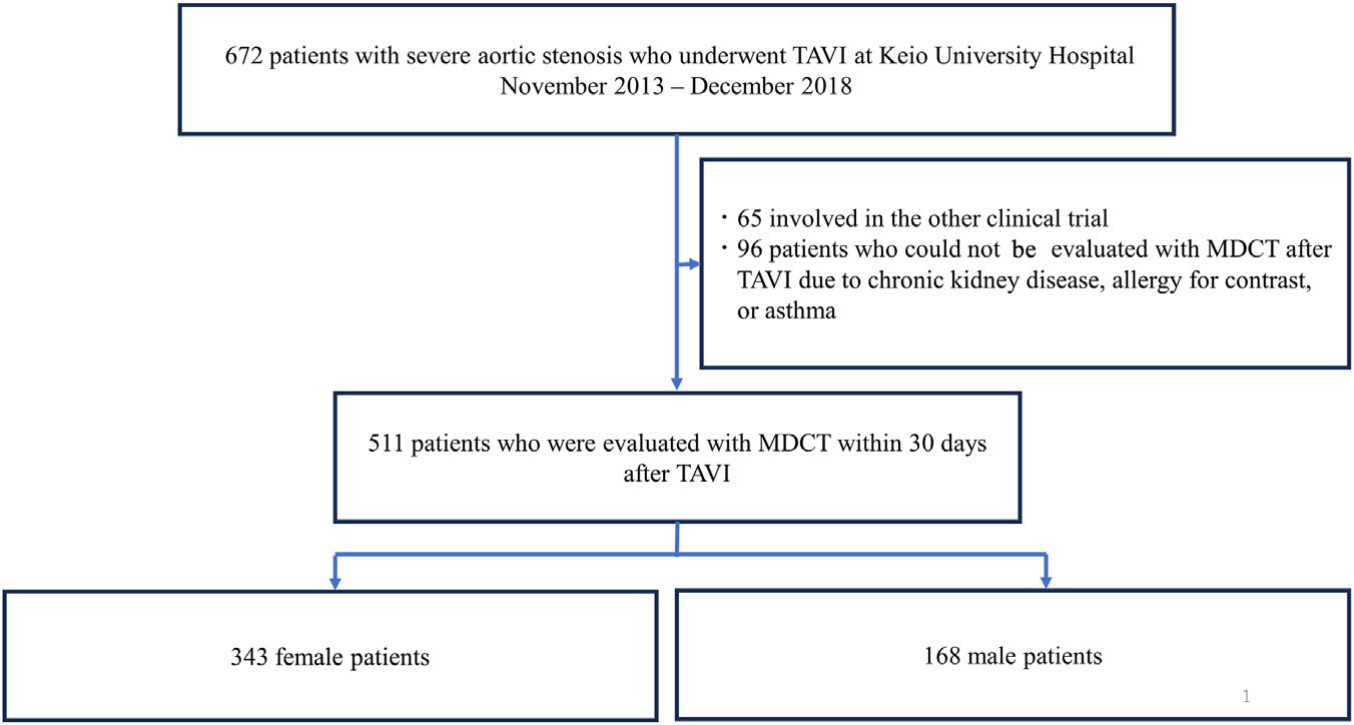
Study cohort. MDCT, multidetector computed tomography; TAVI, transcatheter aortic valve implantation.

Among the 672 consecutive patients who underwent TAVI between October 2013 and December 2018 at Keio University Hospital, 511 met the inclusion criteria and were analyzed (Fig. 1). A total of 343 of the 511 patients (67.2%) were women. The baseline characteristics of the study cohort are presented in Table 1. Baseline characteristics differed significantly between women and men. Women were older at the time of TAVI (mean age: 84.8 ± 5.3 vs 83.8 ± 5.3 years, *P* = 0.025). They had a smaller body size (body mass index: 21.9 ± 3.7 vs 22.8 ± 3.2 kg/m^2^, *P* = 0.002), a greater degree of frailty (clinical frailty scale score ≥ 4: 53.9% vs 32.1%, *P* < 0.001), more-significant shortness of breath (New York Heart Association class ≥ 3: 42.6% vs 31.5%, *P* = 0.016), higher brain natriuretic peptide levels (230 [114-493] vs 165 [70-363] pg/mL, *P* = 0.001), lower hemoglobin levels (11.1 ± 1.4 vs 12.1 ± 1.7 g/dL, *P* < 0.001), and a higher Society of Thoracic Surgeons score (6.5% ± 3.4% vs 5.8% ± 3.6%, *P* = 0.002) than did men. Women were less likely to suffer from coronary artery disease (30.0% vs 45.8%, *P* < 0.001), chronic obstructive pulmonary disease (11.4% vs 26.2%, *P* < 0.001), and carotid artery stenosis (3.8% vs 8.9%, *P* = 0.016) than were men, resulting in less previous history of percutaneous coronary intervention (18.7% vs 31.0%, *P* = 0.002) or stroke (5.0% vs 9.5%, *P* = 0.048).

**Table 1:** Baseline characteristics

Characteristic	Women (n = 343)	Men (n = 168)	*P*
**Preoperative**			
Age, y	84.8 ± 5.3	83.8 ± 5.3	0.025
STS score, %	6.5 ± 3.4	5.8 ± 3.6	0.002
Body mass index, kg/m^2^	21.9 ± 3.7	22.8 ± 3.2	0.002
Clinical frailty scale score ≥ 4	185 (53.9)	54 (32.1)	< 0.001
NYHA class 3 or 4	146 (42.6)	53 (31.5)	0.016
Albumin, g/dL	3.8 ± 0.4	3.9 ± 0.4	0.088
Hemoglobin, g/dL	11.1 ± 1.4	12.1 ± 1.7	< 0.001
BNP, pg/mL (median and interquartile range)	230 (114–493)	165 (70–363)	0.001
**Concomitant disease**			
Hypertension	270 (78.7)	137 (81.5)	0.46
Dyslipidemia	185 (53.9)	93 (55.4)	0.76
Coronary artery disease	103 (30.0)	77 (45.8)	< 0.001
Diabetes mellitus	82 (23.9)	52 (31.0)	0.089
History of atrial fibrillation	70 (20.4)	34 (20.2)	0.96
Chronic kidney disease (GFR< 60 mL/min/1.73 m²)	251 (73.2)	118 (70.2)	0.49
Peripheral artery disease	35 (10.2)	22 (13.1)	0.33
COPD	39 (11.4)	44 (26.2)	< 0.001
Carotid artery stenosis	13 (3.8)	15 (8.9)	0.016
**Previous history**			
Coronary artery bypass grafting	14 (4.1)	12 (7.1)	0.14
Percutaneous coronary intervention	64 (18.7)	52 (31.0)	0.002
Myocardial infarction	11 (3.2)	9 (5.4)	0.24
Stroke	17 (5.0)	16 (9.5)	0.048
Pacemaker	16 (4.7)	9 (5.4)	0.73
**Echocardiographic variables**			
Peak flow velocity, m/s	4.65 ± 0.75	4.50 ± 0.66	0.011
Mean pressure gradient, mm Hg	50.9 ± 18.0	47.5 ± 15.4	0.024
Aortic valve area, cm^2^	0.59 ± 0.16	0.71 ± 0.19	< 0.001
Index aortic valve area, cm^2^/m^2^	0.44 ± 0.11	0.44 ± 0.12	0.58
Ejection fraction, % (Simpson)	62.6 ± 11.3	62.5 ± 11.7	0.92
Moderate or severe AR	26 (7.6)	9 (5.4)	0.35
Moderate or severe MR	35 (10.2)	6 (3.6)	0.01
**MDCT variables**			
Aortic annulus area, mm^2^	365 ± 52	445 ± 61	< 0.001
Area < 430 mm^2^	311 (90.7)	74 (44.0)	< 0.001
Right MLD, mm	6.1 ± 0.9	6.7 ± 1.2	< 0.001
Left MLD, mm	5.9 ± 1.0	6.6 ± 1.2	< 0.001

Values are mean (± standard deviation), or count (%), unless otherwise indicated. *P*-values are from χ^2^ test (n x 2 comparisons) or Student *t*test (continuous parameters). AR, aortic regurgitation; BNP, brain natriuretic peptide; COPD, chronic obstructive pulmonary disease; GFR, glomerular filtration rate; MDCT, multidetector computed tomography; MLD, minimal lumen diameter; MR, mitral regurgitation; NYHA, New York Heart Association; STS, Society of Thoracic Surgeons.

Transthoracic echocardiography demonstrated a higher severity of aortic stenosis (peak flow velocity, 4.65 ± 0.75 vs 4.50 ± 0.66 m/s, *P* = 0.011; aortic valve area, 0.59 ± 0.16 vs 0.71 ± 0.19 cm^2^, *P* ≤ 0.001; MPG, 50.9 ± 18.0 vs 47.5 ± 15.4 mm Hg, *P* = 0.024) and a higher incidence of moderate or severe mitral valve regurgitation, 10.2% vs 3.6%, *P* = 0.01) in women than in men.

MDCT analysis demonstrated that the aortic annulus area was smaller in women than in men (365 ± 52 vs 445 ± 61 mm^2^, *P* < 0.001), and 90.7% of women had a smaller annulus, of < 430 mm^2^.

### Procedural characteristics, complications, and echocardiographic outcomes within 30 days

The procedures and complications are summarized in Table 2. The implanted valve types were not significantly different for women vs men, although the valve size was smaller in women than in men, based on their annulus size. Notably, 20-mm balloon-expandable valves were used in women only.

**Table 2:** Procedure outcomes and complications

Procedure characteristics	Women (n = 343)	Men (n = 168)	*P*
**Type of valve**			
**Balloon-expandable valve**			
SAPIEN XT	133 (38.8)	65 (38.7)	
SAPIEN 3	165 (48.1)	85 (50.6)	
**Self-expandable valve**			
CoreValve	8 (2.3)	3 (1.8)	
Evolut R	31 (9.0)	13 (7.7)	
Evolut PRO	6 (1.7)	2 (1.2)	0.95
**Predilation**	124 (36.2)	63 (37.5)	0.77
**Postdilation**	84 (24.5)	45 (26.8)	0.58
**Valve size, mm**			
0	37 (10.8)	0 (0)	
23	231 (67.3)	44 (26.2)	
26	65 (19.0)	103 (61.3)	
29	10 (2.9)	21 (12.5)	< 0.001
**HALT within 30 d**	52 (15.2)	22 (13.1)	0.53
**Procedural complications**			
**Stroke**	6 (1.7)	2 (1.2)	0.63
Ischemic	6 (1.7)	1 (0.6)	0.29
Hemorrhagic	0 (0)	1 (0.6)	0.15
Disabling	3 (0.9)	1 (0.6)	0.74
Transient ischemic attack	0 (0)	1 (0.6)	0.15
**Bleeding**	21 (6.1)	7 (4.2)	0.36
Minor	10 (2.9)	2 (1.2)	0.23
Major and/or life-threatening	12 (3.5)	5 (3.0)	0.76
**All vascular complications**	49 (14.3)	10 (6.0)	0.006
**New permanent pacemaker**	19 (5.5)	15 (8.9)	0.15
**Hospital stay, d(median and interquartile range)**	9 (7–13)	9 (7–12)	0.08
**Oral anticoagulant therapy**	82 (23.9)	36 (21.4)	0.53
**No antithrombotic therapy**	13 (3.8)	4 (2.4)	0.40

Values are mean (± standard deviation), or count (%), unless otherwise indicated. *P*-values are from the Fisher test (2 x 2 comparison), the χ^2^ test (n x 2 comparisons), or the Student *t* test (continuous parameters). CoreValve, Evolut R, and Evolut PRO are from Medtronic (Minneapolis, MN); SAPIEN XT and SAPIEN 3 are from Edwards Lifesciences (Irvine, CA).

HALT, hypoattenuated leaflet thickening.

MDCT analysis within 30 days after TAVI demonstrated that HALT was detected in 74 patients (14.5%) in total. Women and men showed no significant difference in the incidence of HALT (15.2% vs 13.1%, *P* = 0.53).

The incidence of vascular complications was significantly higher in women than in men (14.3% vs 6.0%, *P* = 0.006). No significant differences were observed in the incidence of other procedural complications, including ischemic and/or hemorrhagic and/or disabling and/or transient ischemic stroke, major or life-threatening bleeding, or new permanent pacemaker implantation.

The postprocedural echocardiographic data are shown in Table 3. The peak flow velocity, EOA, and MPG were less favourable in women than they were in men, for each type of balloon- and self-expandable valve. Hemodynamics were well improved in both genders after TAVI (for balloon-expandable valves—peak flow velocity, 2.45 ± 0.46 vs 2.26 ± 0.41 m/s, *P* < 0.001; EOA, 1.45 ± 0.35 vs 1.77 ± 0.45 cm^2^, *P* < 0.001; MPG, 12.6 ± 5.5 vs 10.3 ± 4.0 mm Hg, *P* < 0.001; for self-expandable valves—2.24 ± 0.49 vs 1.91 ± 0.37 m/s, *P* = 0.011; 1.55 ± 0.34 vs 1.91 ± 0.49 cm^2^, *P* = 0.004; 9.9 ± 4.6 vs 7.3 ± 3.1 mm Hg, *P* = 0.009). However, no significant difference occurred in the indexed EOA. Furthermore, women and men showed no significant difference in incidence of PPM.

**Table 3:** Echocardiographic outcomes at discharge

Measure	Balloon-expandable valve			Self-expandable valve		
	Women (n = 298)	Men (n = 150)	*P*	Women (n = 45)	Men (n = 18)	*P*
**Discharge or postprocedure**						
Peak flow velocity, m/s	2.45 ± 0.46	2.26 ± 0.41	< 0.001	2.24 ± 0.49	1.91 ± 0.37	0.011
Effective orifice area, cm^2^	1.45 ± 0.35	1.77 ± 0.45	< 0.001	1.55 ± 0.34	1.91 ± 0.49	0.004
Index effective orifice area, cm^2^/m^2^	1.08 ± 0.27	1.11 ± 0.31	0.42	1.20 ± 0.32	1.19 ± 0.28	0.99
Prosthetic valve mean gradient, mm Hg	12.6 ± 5.5	10.3 ± 4.0	< 0.001	9.9 ± 4.6	7.3 ± 3.1	0.009
Paravalvular regurgitation						
None or trace	144 (48.3)	61 (40.7)		9 (20.0)	3 (16.7)	
Mild	154 (51.7)	88 (58.7)		34 (75.6)	13 (72.2)	
Moderate	0 (0)	1 (0.7)	0.13	2 (4.4)	2 (11.1)	0.61
Prosthesis–patient mismatch						
Insignificant	242 (81.8)	121 (80.7)		42 (93.3)	17 (94.4)	
Moderate	46 (15.5)	24 (16.0)		1 (2.2)	1 (5.6)	
Severe	8 (2.7)	5 (3.3)	0.92	2 (4.4)	0 (0)	0.54

Values are mean (± standard deviation), or n (%). *P*-values are from the Fisher test for the SAPIEN XT (Edwards Lifesciences, Irvine, CA), the χ^2^ test for the SAPIEN 3 (Edwards Lifesciences), or the Student *t* test (continuous parameters).

### Long-term clinical outcomes

**Figure 2:**
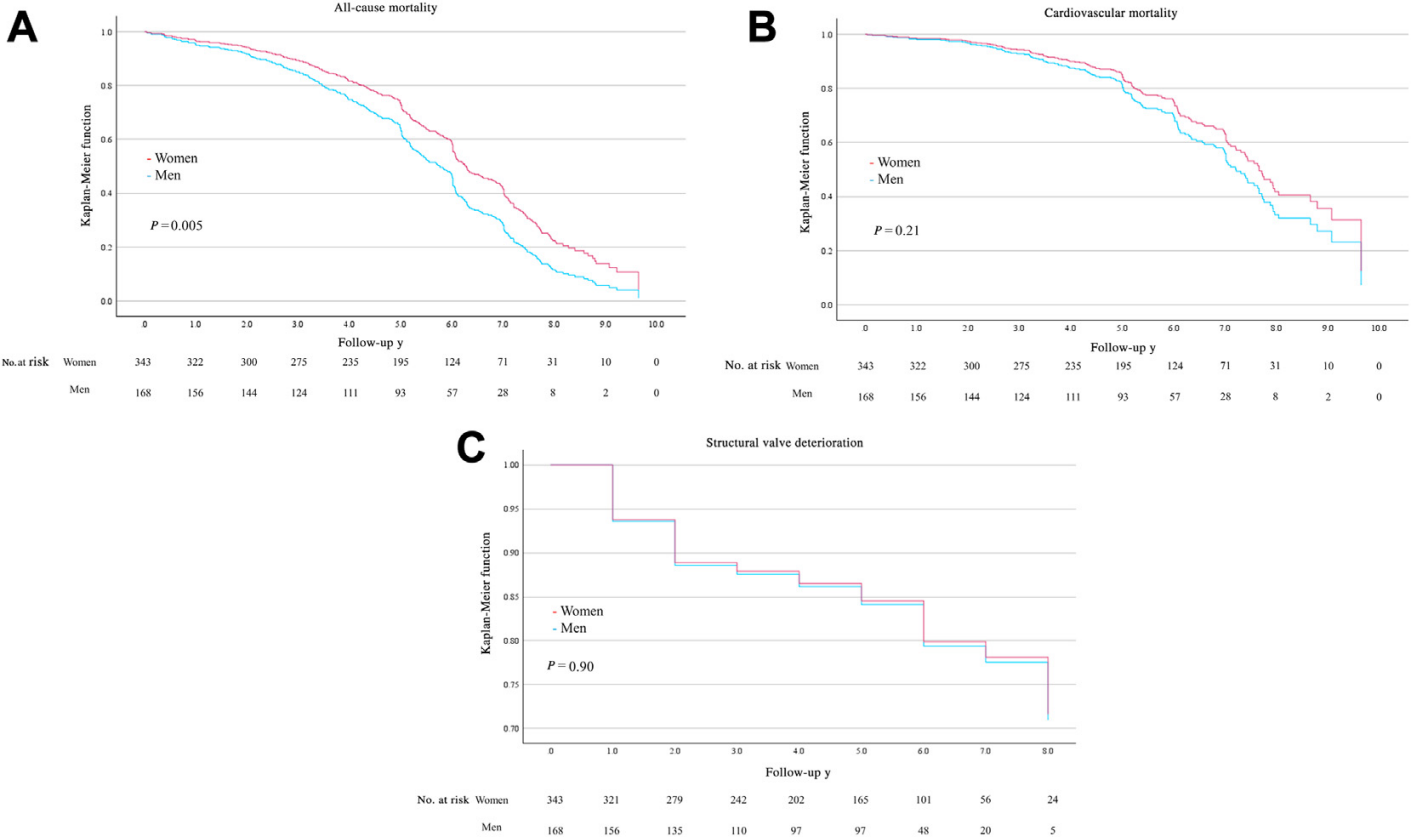
Kaplan–Meier curves of long-term clinical outcomes: (**A**) all-cause mortality; (**B**) cardiovascular mortality; (**C**) structural valve deterioration.

The median follow-up duration was 1844 days (interquartile range, 1190-2311 days), and the long-term clinical outcomes are presented in Table 4. The Kaplan–Meier curves depicting the incidences of all-cause mortality, cardiovascular death, and SVD, according to gender, are shown in Figure 2. Throughout the study period, the incidence of all-cause mortality was lower in women than in men (198 [57.7%] vs 106 (63.1%), HR, 0.69, 95% CI, 0.54-0.90, *P* = 0.005), whereas a higher incidence of VARC-3–defined major bleeding (34 [9.9%] vs 6 [3.6%], HR, 2.80, 95% CI, 1.08-7.23; *P* = 0.03) was noted in women. No differences were observed in the incidences of other outcomes for women vs men, including cardiovascular death, ischemic stroke, hemorrhagic stroke, heart failure rehospitalization, or SVD (Table 4). Among patients for whom detailed cause-of-mortality records are available, for cardiovascular death, heart failure was the most common cause, followed by infective endocarditis. For noncardiovascular death, pneumonia, including aspiration pneumonia, was the most common cause, followed by malignancy. SVD consists of stenosis and regurgitation, and the mode of failure, by gender, was listed as follows: 48.7% of women, and 37.1% of men, had the etiology of stenosis, and the rest developed regurgitation. The incidence of SVD in each valve type was as follows: for balloon-expandable valves—52 of 298 (17.4%) in women, and 25 of 150 (16.7%) in men; for self-expandable valves—3 of 45 (6.7%) in women, and 4 of 18 (22.2%) in men.

**Table 4:** Long-term clinical outcomes

Outcome	Women (n = 343)	Men (n = 168)	HR (95% CI)	*P*
**All-cause death**	198 (57.7)	106 (63.1)	0.69 (0.54–0.90)	0.005
Cardiovascular death	111 (32.4)	48 (28.6)	0.79 (0.55–1.14)	0.21
**Structural valve deterioration**	55 (16.0)	29 (17.3)	0.97 (0.60–1.57)	0.90
**Stroke**				
Ischemic	17 (5.0)	9 (5.4)	0.77 (0.31–1.94)	0.59
Hemorrhagic	12 (3.5)	2 (1.2)	2.59 (0.54–12.4)	0.23
**VARC-3 major bleeding**	34 (9.9)	6 (3.6)	2.80 (1.08–7.23)	0.03
**Heart failure readmission**	49 (14.3)	21 (12.5)	0.98 (0.57–1.69)	0.94

Values are count (%), unless otherwise indicated. *P*-values are from the Cox proportional hazards model. The hazard ratio (HR) shown is for women with respect to men. CI, confidence interval; VARC-3, Valve Academic Research Consortium-3.

**Figure 3:**
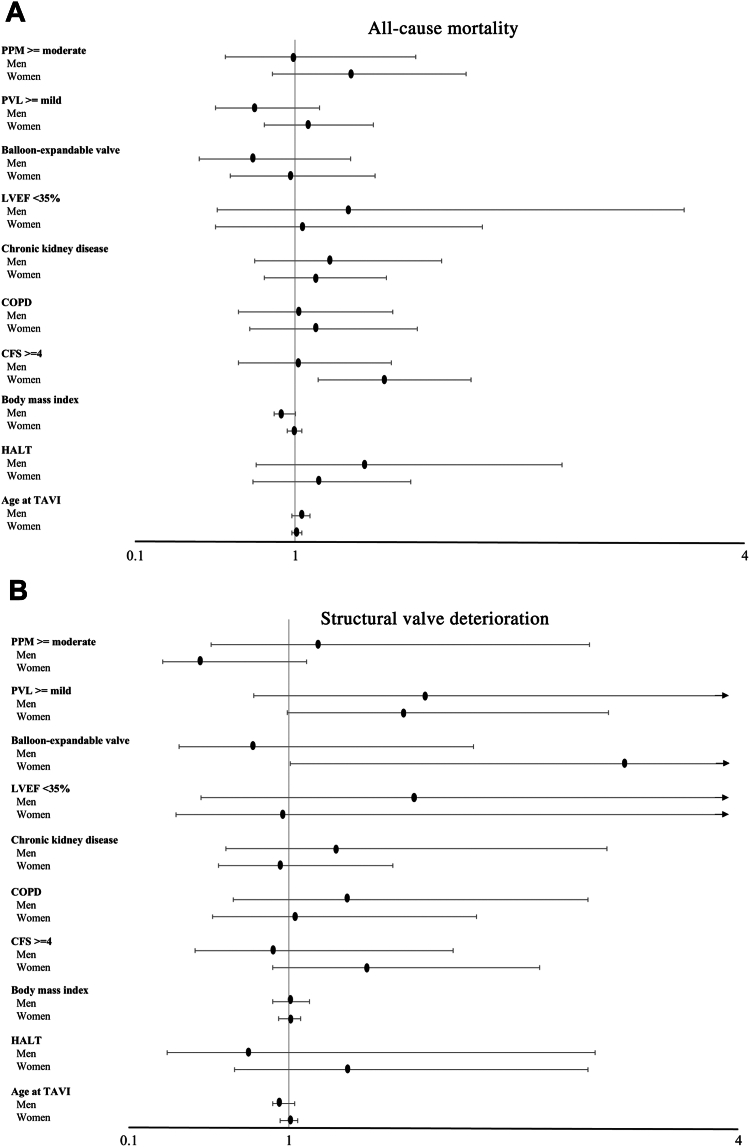
Forest plot of independent predictors for all-cause mortality and structural valve deterioration: (**A**) all-cause mortality; (**B**) structural valve deterioration. CFS, clinical frailty scale; COPD, chronic obstructive pulmonary disease; HALT, hypoattenuated leaflet thickening; LVEF, left ventricular ejection fraction; PPM, prosthesis–patient mismatch; PVL, paravalvular leakage; TAVI, transcatheter aortic valve implantation.

The independent predictors of all-cause mortality and SVD according to gender are presented in Figure 3. In multivariable analysis, the incidence of all-cause mortality in women was associated independently with a clinical frailty scale score of > 4 (HR, 1.54, 95% CI, 1.14-2.08, *P* = 0.005). The balloon-expandable valves were associated independently with the development of SVD in women (HR, 3.28, 95% CI, 1.01-10.67, *P* = 0.049) but not in men. SVD, based on the definition used in this study, was not associated with all-cause mortality in either men or women.

## Discussion

To our knowledge, the present study is the first to evaluate gender differences in long-term outcomes with a median time period of > 5 years. In this study, women were approximately 67.2% of all patients who received the TAVI procedures. The salient findings of the present analysis, comparing women and men, can be summarized as follows:

• As baseline characteristics, women had smaller anatomic aortic complexes and a higher severity of aortic stenosis than did men.
• Women had more vascular complications than did men.
• Although women have a smaller annulus and a higher severity of aortic stenosis preoperatively, the incidence of PPM and HALT on MDCT did not differ based on gender.
• Women had favourable long-term all-cause mortality, but they were more likely to suffer from major bleeding.
• More than 5 years after TAVI, the incidence of development of SVD was comparable for women vs men.

Gender differences in prognosis after TAVI up to the midterm point have been studied, and women showed better prognosis after TAVI than did men.^6^, ^7^, ^8^, ^9^ However, conclusions regarding long-term prognostic outcomes and the incidence of SVD remain unclear.

In this study, 90% of the women had a small annulus, meaning the area was < 430 mm^2^. Notably, the eligible patients in the current study, the Asian population, tended to have a smaller aortic annulus, compared with the size in previous studies with Westerners, which demonstrated that 82.5% of women had a small annulus.^11^ In this study, when a conversion was made to the index aortic valve area, no significant differences were observed between genders. Furthermore, in the balloon- and self-expandable valves, the incidence of PPM, which was calculated based on body size, was comparable between the genders. Previous studies also have reported that women are more likely to develop PPM than are men^11^; however, the smaller body surface area in this study must have favoured avoidance of PPM in the small annulus.

Vascular complications were more common in women than in men in this study, consistent with results of previous reports.^6^^,^^12^ Unavoidable differences are that women have significantly smaller vascular diameters than those in men, and vascular complications are more common in women than in men. However, to prevent that kind of complication as much as possible, careful device selection and attention at the time of final closure are crucial. In the long term, women had more bleeding events than did men. We speculate that one of the reasons for this phenomenon, when taking into account that no gender difference occurred in the rate of anticoagulant medication, may be that lower body weight generally is more common in women, especially elderly Japanese women, and the degree of frailty also is higher in women than it is in men in this study cohort.

The advantage of this study was that MDCT was performed within 30 days after TAVI was analyzed, to detect early subclinical HALT in all patients. However, whether the incidence of HALT is associated with clinical outcomes, in either gender, has not been determined clearly.^13^^,^^14^ In this study, no gender differences were observed in the incidence of early HALT (15.2% vs 13.2%, *P* = 0.53). Our previous study found that the early detection of HALT was not associated with medium-term prognosis.^13^^,^^15^ However, the present study suggests that, in the long term, HALT is not associated with the prognosis or incidence of SVD in women and men. Generally, patients who underwent TAVI were older and were more likely to be at high risk of bleeding, especially women.^12^ Women had more bleeding events in the long term in this study, a result consistent with findings of previous reports (HR, 2.80; 95% CI, 1.08-7.23, *P* = 0.03). Use of additional anticoagulation to treat HALT, in response to indications, should be considered carefully, taking into account the prognostic impact of both SVD and the bleeding event, especially in this elderly cohort.

Our results showed that all-cause mortality was less common in women than in men (HR, 0.69, 95% CI, 0.54-0.90, *P* = 0.005). This difference is due to men having more comorbidities before undergoing TAVI, including coronary artery disease, previous percutaneous coronary intervention, and chronic obstructive pulmonary disease. Women have a longer life expectancy than do men, biologically.^16^ In the current study, women had less comorbidity of coronary artery disease than men did in the patient background, and they had fewer cardiovascular events during long-term follow-up, although this difference was not statistically significant (HR, 0.79, 95% CI, 0.55-1.14, *P* = 0.21). This result suggests that noncardiovascular deaths are more common in men. Given that previous studies have suggested that pulmonary complications are the main reason for fatal outcomes after a TAVI procedure,^17^^,^^18^ the significantly higher prevalence of preoperative chronic obstructive pulmonary disease is considered to contribute to the incidence of noncardiovascular deaths. However, the present study indicated that severe frailty, as indicated by a clinical frailty scale score of > 4, was an independent predictor of a higher incidence of mortality in women. This result suggests that proper frailty assessment before TAVI is performed is important, to improve the long-term prognosis of women.

No difference in the incidence of SVD between women and men was observed during long-term follow-up of echocardiographic data (HR, 0.99, 95% CI 0.78-1.25, *P* = 0.90). Furthermore, paravalvular leakage that remains less than mild immediately after TAVI may be a poor prognostic factor for the development of SVD in both genders, but no such finding was statistically significant. In this study, we also showed that balloon-expandable valves were more likely than self-expandable valves to cause SVD in women in the long term. This finding was similar to the results of the **Sm**all **A**nnuli **R**andomized to Evolut or SAPIEN trial (SMART), which showed that self-expandable valves were less likely to cause SVD than were balloon-expandable valves 12 months after TAVI was performed in patients with a small annulus.^19^ Women of an advanced age who had small annuli showed excellent clinical outcomes without developing SVD, even though SVD itself was not associated with mortality; therefore, an appropriate TAVI procedure would improve the long-term prognosis and lifetime management of younger patients.

This study has some limitations. First, as this was an observational cohort study, our findings may have been influenced by unmeasured variables and should be interpreted with caution. However, the potential for bias owing to confounding factors could not be eliminated entirely, despite the use of sophisticated statistical methods. Second, the number of patients included in this study was relatively small, because the study was conducted at a single centre. However, long-term accurate data on valve performance and large number of MDCT data from within 30 days after TAVI was performed were available in the current study. Third, TAVI procedures performed using former-generation devices were analyzed, because we aimed to investigate the long-term prognosis for or development of SVD. However, the latest valves may have better durability than that of the former ones. Therefore, further studies using newer-generation devices are required. Finally, many patients included in this study were at high risk and were old; thus, only a limited number of patients were able to be followed long term. In the future, the impact of gender differences on the prognosis for or development of SVD should be examined in more patients.

### Conclusion

Among those patients with severe aortic stenosis who underwent TAVI, more than 90% of the women had a small annulus. Women may have superior long-term clinical outcomes. The incidence of SVD in women was similar to that in the entire cohort; however, it was higher in a cohort who received a balloon-expandable valve. The association between gender differences and clinical outcomes should be delineated further in future research, using larger numbers of patients.
